# Improved ROS defense in the swimbladder of a facultative air-breathing erythrinid fish, jeju, compared to a non-air-breathing close relative, traira

**DOI:** 10.1007/s00360-016-0981-5

**Published:** 2016-04-05

**Authors:** Bernd Pelster, Marina Giacomin, Chris M. Wood, Adalberto L. Val

**Affiliations:** Institute of Zoology, University of Innsbruck, Technikerstr. 25, 6020 Innsbruck, Austria; Center for Molecular Biosciences, University Innsbruck, Innsbruck, Austria; Department of Zoology, University of British Columbia, Vancouver, BC V6T 1Z4 Canada; Instituto Nacional de Pesquisas da Amazônia, Manaus, Brazil

**Keywords:** Swimbladder, Erythrinid fish, Reactive oxygen species, ROS defense, Oxygen partial pressure

## Abstract

The jeju *Hoplerythrinus unitaeniatus* and the traira *Hoplias malabaricus* are two closely related erythrinid fish, both possessing a two-chambered physostomous swimbladder. In the jeju the anterior section of the posterior bladder is highly vascularized and the swimbladder is used for aerial respiration; the traira, in turn, is a water-breather that uses the swimbladder as a buoyancy organ and not for aerial oxygen uptake. Observation of the breathing behavior under different levels of water oxygenation revealed that the traira started aquatic surface respiration only under severe hypoxic conditions and did not breathe air. In the jeju air-breathing behavior was observed under normoxic conditions, and the frequency of air-breathing was significantly increased under hypoxic conditions. Unexpectedly, even under hyperoxic conditions (30 mg O_2_ L^−1^) the jeju continued to take air breaths, and compared with normoxic conditions the frequency was not reduced. Because the frequently air-exposed swimbladder tissue faces higher oxygen partial pressures than normally experienced by other fish tissues, it was hypothesized that in the facultative air-breathing jeju, swimbladder tissue would have a higher antioxidative capacity than the swimbladder tissue of the water breathing traira. Measurement of total glutathione (GSSG/GSH) concentration in anterior and posterior swimbladder tissue revealed a higher concentration of this antioxidant in swimbladder tissue as compared to muscle tissue in the jeju. Furthermore, the GSSG/GSH concentration in jeju tissues was significantly higher than in traira tissues. Similarly, activities of enzymes involved in the breakdown of reactive oxygen species were significantly higher in the jeju swimbladder as compared to the traira swimbladder. The results show that the jeju, using the swimbladder as an additional breathing organ, has an enhanced antioxidative capacity in the swimbladder as compared to the traira, using the swimbladder only as a buoyancy organ.

## Introduction

The oxygen concentration in aquatic systems, in particular in the Amazon Basin, is known to vary greatly (Val and Almeida-Val 1995; Muusze et al. 1998; Diaz and Breitburg 2009; Welker et al. 2013). Oxygen is essential for aerobic metabolism and ATP production, and reduced oxygen availability frequently results in metabolic depression (St-Pierre et al. 2000; Guppy 2004; van Ginneken and van den Thillart 2009; Ali et al. 2012). To supplement oxygen supply during aquatic hypoxia, many fish of the Amazon Basin rely on aquatic surface respiration or even use specific tissues or organs for aerial gas exchange (Val and Almeida-Val 1995). For example, members of the Loricariidae and the Callichthyidae families frequently use their vascularized stomach or intestine for aerial gas exchange, the tambaqui picks up oxygen from the water surface with a largely extended inferior lip under hypoxic conditions, and some species of the Erythrinidae family of fish use the swimbladder to extract oxygen from the air. Pharyngeal, branchial and mouth diverticula are found among the Electrophoridae and the Synbranchidae, while the South-American lungfish *Lepidosiren paradoxa* takes most of the oxygen required for aerobic metabolism using its well-developed lung (Bemis et al. 1987; Val and Almeida-Val 1995; Glass and Rantin 2009).

While oxygen is essential for aerobic metabolism, it may also result in the production of reactive oxygen species (ROS). ROS are mainly generated by electrons escaping from the mitochondrial electron transport chain and by NADPH-oxidase. To a minor extent the endoplasmic reticulum with cytochrome P450 and different cellular oxidases may also contribute to ROS production (Chandel and Budinger 2007; Lushchak 2011). The accumulation of ROS causes oxidative stress (Lushchak 2014; Sies 2015) and results in lipid peroxidation, protein carbonylation, and/or DNA modifications, i.e., formation of oxidized bases, in particular 8-oxoguanine (Lushchak 2011). In consequence, the inordinate accumulation of ROS causes serious tissue damage and is harmful for the whole organism.

Hyperoxia stimulates the generation of ROS, and hyperoxic conditions have been shown to result in transient oxidative stress in goldfish tissues (Lushchak et al. 2005a; Lushchak and Bagnyukova 2006; Lushchak 2011). Normoxic recovery following transient exposure to hypoxic conditions also sharply increases the ROS level, resulting in oxidative stress (Halliwell and Gutteridge 1989; Li and Jackson 2002; Hermes-Lima 2004; Gorr et al. 2010). Meanwhile it has been shown repeatedly that reduced oxygen availability (hypoxia) may also cause oxidative stress (Welker et al. 2013), which in fish occurs, for example, in goldfish *Carassius auratus* (Lushchak et al. 2001), common carp *Cyprinus carpio* (Lushchak et al. 2005b), rotan *Perccottus glenii* (Lushchak and Bagnyukova 2007), and the Indian catfish *Clarias batrachus* (Tripathi et al. 2013).

These considerations show that ROS are frequently expected in nature with changing oxygen availability and in tissues exposed to high PO_2_, and organisms have developed sophisticated defense systems to break down and detoxify reactive oxygen molecules (Hermes-Lima 2004; Lushchak 2011; Summarwar and Verma 2012; Welker et al. 2013; Lushchak 2014). These can include the accumulation of antioxidants like glutathione, ascorbic acid (vitamin C), retinol (vitamin A) or α-tocopherol (vitamin E). In addition, several enzymes are able to remove ROS, and these include catalase, superoxide dismutase (SOD), glutathione reductase (GR) and glutathione peroxidase (GPx). Glucose-6-phosphate dehydrogenase may also be mentioned in this context. This enzyme contributes to the pentose phosphate shunt and generates NADPH, which is required for the regeneration of GSH in the GR reaction.

The concentration of antioxidants and the activity of enzymatic antioxidants have been analyzed in many tissues and several species of fish under different environmental stress conditions [see (Lushchak and Bagnyukova 2006; Lushchak 2011; Welker et al. 2013)]. These studies clearly showed that the defense system against ROS is not static but highly responsive to changing environmental conditions. A significant increase in ROS degrading enzyme activities and in the concentration of antioxidants is, for example, frequently observed following periods of hypoxia. Hermes-Lima et al. (1998) proposed that hypoxia prepares the organism for oxidative stress encountered during recovery from hypoxia, and the concept of a preparation for oxidative stress is widely accepted (Welker et al. 2013).

Particularly high activities of enzymes involved in ROS degradation are typically found in tissues prone to be exposed to ROS like the liver (Lushchak et al. 2005a; Bagnyukova et al. 2006), or the lung in mammals, which faces the highest oxygen concentrations in mammalian tissues (Erzurum et al. 1993; Ho et al. 2001; Hackett et al. 2003). In marine fish high activities are also found in swimbladder tissue (Morris and Albright 1981, 1984), because the swimbladder of physoclist fish typically contains high concentrations of oxygen and with increasing hyperbaric pressure at depth becomes extremely hyperoxic (Pelster 1997, 2011, 2015). PO_2_ in the swimbladder of fish breathing air cannot be as high as in fish swimming at depth, but they certainly are higher than in most other tissues, except for gills and skin, because arterial oxygen partial pressure of water breathing fish typically is much lower than aerial PO_2_ (Gilmour and Perry 1994; Kristensen et al. 2010). PO_2_ in the physostomous swimbladder of fish using the swimbladder for breathing air should also be higher than in fish using the swimbladder only as a buoyancy structure. In fish breathing air, the swimbladder gas is renewed frequently with fresh air, but in fish using the bladder as a buoyancy structure, fresh air is engulfed only occasionally. In the intervening period, however, gases are resorbed according to their physical solubility, and this means that nitrogen is accumulated in the swimbladder, replacing oxygen (Piiper et al. 1962; Piiper 1965). We therefore hypothesized that the swimbladder tissue of a freshwater fish using the swimbladder for aerial respiration would be equipped with an enhanced ROS defense system compared with a swimbladder of a closely related fish where the swimbladder is used only as a buoyancy organ, and with other fish tissues.

To test this hypothesis we examined two closely related erythrinid fish, the jeju, *Hoplerythrinus unitaeniatus*, and the traira, *Hopliasmalabaricus.* Both fish have a swimbladder consisting of an anterior and a posterior chamber, connected to the esophagus via a ductus pneumaticus. In the facultative air-breathing jeju the anterior section of the posterior swimbladder is highly vascularized, and it is used for aerial gas exchange. Previous studies have indeed shown that swimbladder PO_2_ in the jeju may even reach 20 kPa in the anterior bladder (Kramer 1978), and Farrell and Randall (1978) estimated a value of about 18 kPa. In traira, neither the anterior nor the posterior part of the bladder shows a dense vascularization and the bladder is likely used as a buoyancy organ, but not for aerial gas exchange (Val and Almeida-Val 1995).

## Materials and methods

All experiments were performed in December 2013 and December 2014 on board a research vessel (the Anna Clara, from Manaus) during two expeditions to the Anavilhanas Archipelago of the Rio Negro, approximately 110 km upstream from Manaus. All procedures were in compliance with Brazilian national and Instituto Nacional de Pesquisas da Amazônia (INPA) animal care regulations.

Erythrinid fish used for this study, the jeju and the traira, were caught by INPA fishermen. Body mass of jeju used for these experiments was 160 ± 23 g, fork length 23.1 ± 1.2 cm (*N* = 15) and of traira 147 ± 18 g, fork length 25.3 ± 1.2 cm (*N* = 14). Both species were held on board in large tanks served with flowing “black water” pumped directly from the Rio Negro (temperature = 30–35 °C, pH = 4.0–4.5). These values are normal for this river, which supports an abundant fish fauna adapted to these extreme conditions (Val and Almeida-Val 1995). In the tanks the fish had free access to air and could therefore breathe air voluntarily. Fish were not fed during the 12-day expeditions and were allowed to recover at least overnight after capture before experimentation.

### Behavioral studies

For behavioral studies individual fish were transferred into individual cylindrical 25–30 L glass containers, filled with Rio Negro water (temperature = 30–35 °C, pH = 4.0–4.5). The differences in river water temperature were encountered between different days during the field trip depending on the weather conditions. However, within an individual experiment temperature did not vary by more than 2 °C. The glass containers were separated from other tanks so that the fish could not see each other, and the fish were allowed to settle for at least 1 h. After this time fish never showed any signs of stress or increased swimming activity. The water surface was uncovered and the fish could take air breaths completely unhindered. The water was aerated using air stones (normoxia = 7–8 mg O_2_ L^−1^, corresponding to a PO_2_ of 20 kPa), or gassed with oxygen to achieve hyperoxic conditions (30 mg O_2_ L^−1^, corresponding to a PO_2_ of 80 kPa). Hypoxic conditions were achieved by gassing the water with nitrogen until an oxygen content of about 0.5 mg O_2_ L^−1^ (corresponding to a PO_2_ of 1.3 kPa) was reached. Treatments were assigned randomly to 10 jeju and 11 traira. Water oxygen concentration and partial pressure were recorded with a portable oxygen electrode and meter (WTW Oxi325 Oximeter, Weilheim, Germany). The air-breathing behavior of each fish was observed for up to 4 h between 10 am and 6 pm, and sequences were recorded using a Nikon D7100 camera. The number of air-breaths taken per unit of time was counted for the jeju. Air-breaths could easily be identified because immediately after leaving the surface air bubbles left the opercular cavity via the operculum. For traira, the frequency of aquatic surface respiration (ASR) was recorded. In addition, the % of time spent at the surface was calculated for both species by measuring the time required for each air-breathing event (jeju) or for aquatic surface respiration ASR (traira) (Kramer and Mehegan 1981) and dividing it by total observation time.

### Tissue preparation

Fish maintained under normoxic conditions were killed with an overdose of tricaine methanesulfonate **(**MS222; 0.3 g L^−1^). Fish were opened ventrally and the swimbladder was carefully dissected. Connective tissue was removed and the remaining tissue was carefully rinsed and cleaned in Cortland saline (Wolf 1963) and blotted dry. The anterior swimbladder tissue of jeju and traira and also the posterior part of traira swimbladder was dissected into small portions and immediately frozen in liquid nitrogen. For jeju, only the vascularized (first) part of the posterior swimbladder tissue was used for tissue preparation. Pieces of white muscle tissue were taken close to the lateral line near the anus and immediately frozen in liquid nitrogen. Tissues were then stored in a biofreezer at −80 °C until further analysis.

### Biochemical analysis

For determination of total glutathione (GSSG/GSH) content of the frozen tissue samples, tissue extracts were prepared using 5 % metaphosphoric acid (MPA). The frozen tissues were ground to a fine powder and dissolved 1:5 w/v in 5 % MPA. Under ice cooling the solution was homogenized using a motorized homogenizer. Extracts were centrifuged at 13,000 rpm for 5 min at 4 °C and the supernatant was diluted using assay buffer for GSSG/GSH determination. GSSG/GSH concentration was determined using the OxiSelect Total Glutathione (GSSG/GSH) Assay Kit (STA-312) Cell Biolabs, Inc, San Diego, USA, following the manufacturer’s instructions.

For measurement of enzyme activities the frozen tissue samples were homogenized on ice in 1:5 w/v of ice-cold homogenization buffer (10 mM TRIS/HCl, 0.1 mM disodium EDTA, 150 mM NaCl, pH 7.5 at 25 °C). Under ice cooling, the solution was homogenized using a motorized homogenizer. Homogenates were centrifuged at 13,000 rpm for 15 min at 4 °C and appropriate dilutions of the supernatant were used for the enzyme and protein assays.

Enzyme activities were measured using a SpectraMax 384Plus microplate spectrophotometer (Molecular Devices, Sunnyvale, CA, USA) with temperature control at 25 ± 0.1 °C. Glutathione reductase (GR; EC 1.6.4.2.) and glutathione peroxidase (GPx; EC 1.11.1.9.) activity was measured using the Glutathione Reductase Assay Kit (No 703202; Cayman Chemical Company, Ann Harbor, USA), and the Glutathione Peroxidase Assay Kit (No 703102; Cayman Chemical Company, Ann Harbor, USA).

Catalase (Cat; EC 1.11.1.6.) activity was assayed using the Amplex Red Catalase Assay Kit (A22180; Molecular Probes, Eugene, USA). Superoxide dismutase (SOD; EC 1.15.1.1.) activity was measured following a procedure described by (McCord and Fridovich 1969). Briefly, reactive oxygen species generated from xanthine in the xanthine oxidase reaction cause a reduction of cytochrome *c*, which is inhibited by the presence of SOD. One unit of SOD activity is defined as the amount of enzyme (per milligram of protein) that inhibits the reduction of cytochrome *c* observed in the blank without SOD by 50 %.

Protein concentration in the homogenate was measured with Coomassie Brilliant Blue G-250 (Bradford 1976) using bovine serum albumin as a standard.

### Statistics

Data have been expressed as mean ± 1 SEM with *N* giving the number of animals analyzed in each species. GSSG/GSH concentrations are given as µmol g^−1^wwt (wet weight), enzyme activities as U mg^−1^protein (µmol min^−1^ mg^−1^ protein). For statistical analysis two-way repeated measures Anova, followed by the Holm-Sidak multiple comparison procedures, was used. Fish species (jeju, traira) and tissue (muscle, anterior bladder, posterior bladder) were used as parameters (factors) 1 and 2, and enzyme activity or GSH/GSSG concentration as variables (data). Air-breathing frequency and % time spent at the surface were analyzed using one-way Anova followed by Tukey’s post hoc test. In rare cases the normality test or equal variance test failed using the original data. In this case the data were log transformed prior to statistical analysis. The statistical analysis was performed using SigmaPlot 12.0. Statistical differences between values were accepted for *p* < 0.05.

## Results

**Fig. 1:**
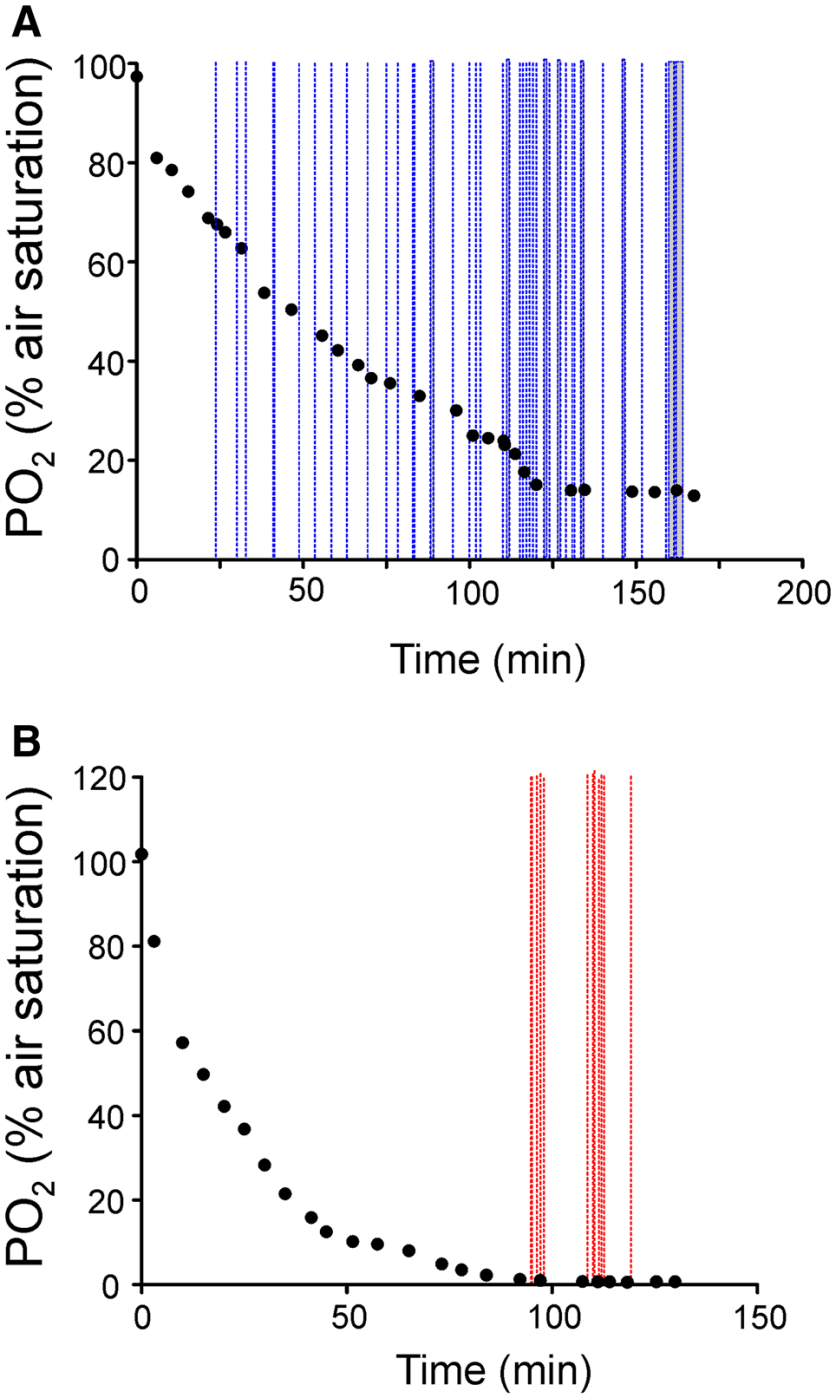
**A** Air-breathing activity in the jeju in an individual experiment with progressive hypoxia. Each *dashed blue line* indicates a single air-breath. Air-breathing frequency was significantly elevated under hypoxic conditions. **B** Aquatic surface respiration activity (*ASR*) in an individual traira. In traira air-breathing was never observed. Under severe hypoxia traira started ASR. Each *dashed red line* indicates a single ASR event. *Black dots* indicate individual oxygen measurements documenting the declining PO_2_.Observations ended after the final PO_2_ measurement (color figure online)

**Fig. 2:**
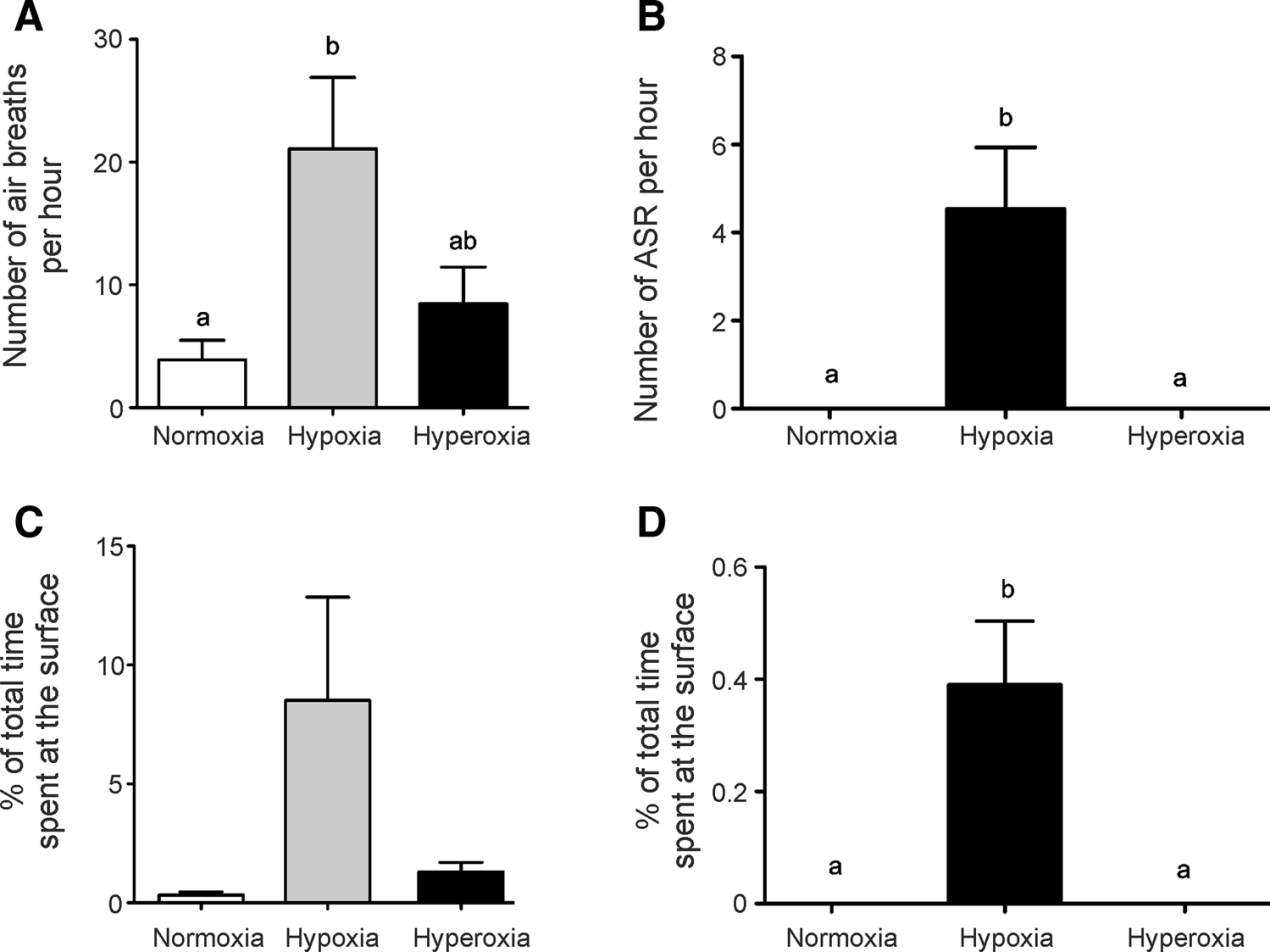
Quantitative analysis of the air-breathing and ASR activity of jeju (*N* = 10) and traira (*N* = 9). **A** Number of air-breaths per hour in jeju under normoxic, hypoxic and hyperoxic conditions. **B** Number of ASR events per hour in traira under normoxia, hypoxia and hyperoxia. **C**, **D** % of time spent at the surface for air-breathing or ASR in jeju (**C**) and traira (**D**), respectively. Small letters denote significant differences between normoxia, hypoxia and hyperoxia (*p* < 0.05). *Bars* without letters are not different from each other

Behavioral observation confirmed that the jeju is a facultative air-breather, repeatedly visiting the surface to take an air-breath under normoxic conditions. With decreasing PO_2_ in the water the frequency of air-breathing increased, as shown for an individual jeju in Fig. 1A. Surprisingly, in the jeju this air-breathing behavior was also routinely observed under hyperoxic conditions (Fig. 2). Air-breathing was never observed in traira, and this species did not visit the water surface for aquatic surface respiration (ASR) under either normoxia or hyperoxia. However, ASR was observed when water PO_2_ was below 5 % of air saturation, i.e., close to zero, as shown for an individual fish in Fig. 1B. The fish swam to the water surface opening the jaws just beneath the surface. However, in the traira the release of an air bubble from the gill cavity was never observed.

Quantitative analysis of air-breathing behavior (jeju) and of ASR (traira) is shown in Fig. 2. Under normoxic conditions the jeju spent close to 1 % of the observed time at the surface breathing air, and under hyperoxic this air-breathing activity was not reduced. Under hypoxic conditions the jeju visited the surface 21.7 times per hour and spent 8.6 % of the time for surface respiration, which was significantly higher than under normoxic or hyperoxic conditions (Fig. 2A, C). The traira in turn started aquatic surface respiration (ASR) under hypoxic conditions and visited the surface 4.4 times per hour, spending about 0.4 % of the time at the surface (Fig. 2B, D). Accordingly, hypoxia significantly stimulated air-breathing behavior (jeju) and ASR (traira), respectively, in the two species.

**Fig. 3:**
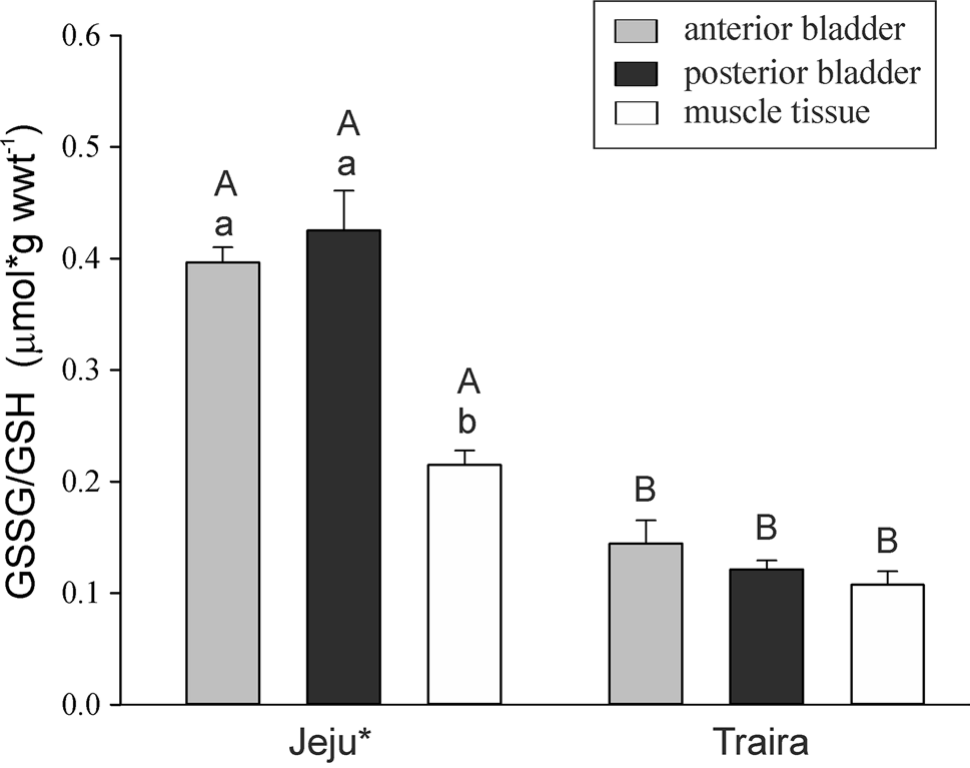
Total GSSG/GSH concentration in anterior and posterior swimbladder tissue and in muscle tissue of jeju and of traira. *Small letters* denote significant differences between tissues within a species, *capital letters* denote significant differences between the two species (*N* = 6; *p* < 0.05)

To assess the antioxidative capacity of swimbladder tissue we measured total glutathione concentration (GSSG/GSH) in the anterior and in the posterior swimbladder tissue, including also muscle tissue for comparison as a tissue that is not directly exposed to high oxygen partial pressures (Fig. 3). A comparison of the two erythrinid species revealed a significantly higher GSSG/GSH concentration in all examined tissues of the jeju, and two-way RM Anova revealed differences between the two species (*p* < 0.001, *F*_1,9_ = 203.328). In the jeju swimbladder in both sections the concentration of total GSSG/GSH was significantly higher as compared to muscle tissue (*p* < 0.001 for anterior versus muscle and for posterior versus muscle; *p* = 0.32 for anterior versus posterior) (Fig. 3). In traira no significant difference was detected between swimbladder tissue and muscle tissue (*p* > 0.1 for all comparisons).

**Fig. 4:**
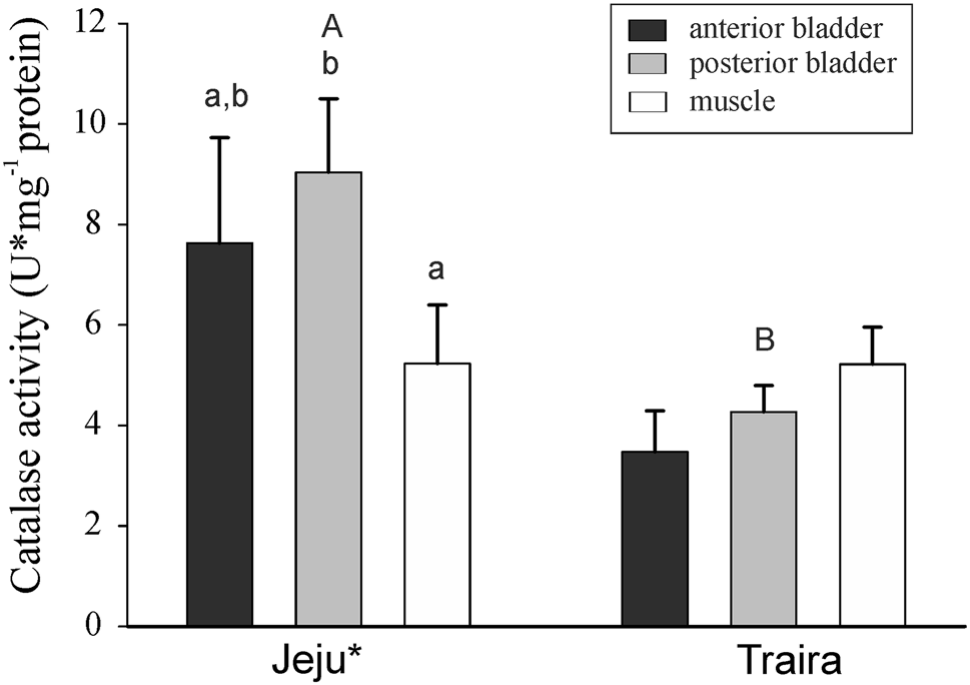
Catalase activity in U mg^−1^ protein in anterior and posterior swimbladder tissue and in muscle tissue of jeju and of traira. *Capital letters* denote significant differences between the two species, *bars* without letters are not significantly different;* asterisk* indicates significant overall difference between the two species (jeju, *N* = 11; traira, *N* = 9; *p* < 0.05)

At the species level catalase activity was higher in jeju (*p* < 0.05; *F*_1,14_ = 6.007), and in posterior swimbladder tissue of jeju catalase activity was higher than in posterior swimbladder tissue of traira (*p* < 0.01) (Fig. 4). Comparison of swimbladder and muscle tissue of jeju revealed higher catalase activity in posterior swimbladder tissue as compared to muscle tissue (*p* < 0.01). No difference in catalase activity was detected among the three traira tissues analyzed.

**Fig. 5:**
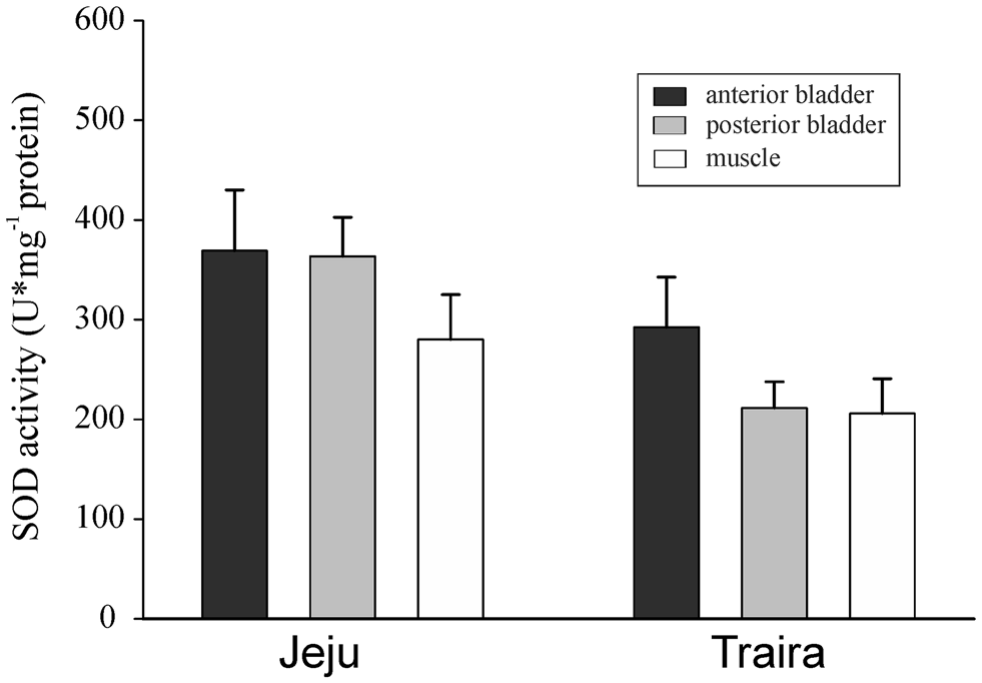
SOD activity in U mg^−1^ protein in anterior and posterior swimbladder tissue and in muscle tissue of jeju and of traira. *Capital letters* denote significant differences between the two species, *bars* without letters are not significantly different (jeju, *N* = 11; traira, *N* = 10; *p* < 0.05)

SOD activity was very similar in the tissues of jeju and traira, and there was no significant difference between the two species (Fig. 5). Similarly, no significant difference was detected among the different tissues of either jeju or traira.

**Fig. 6:**
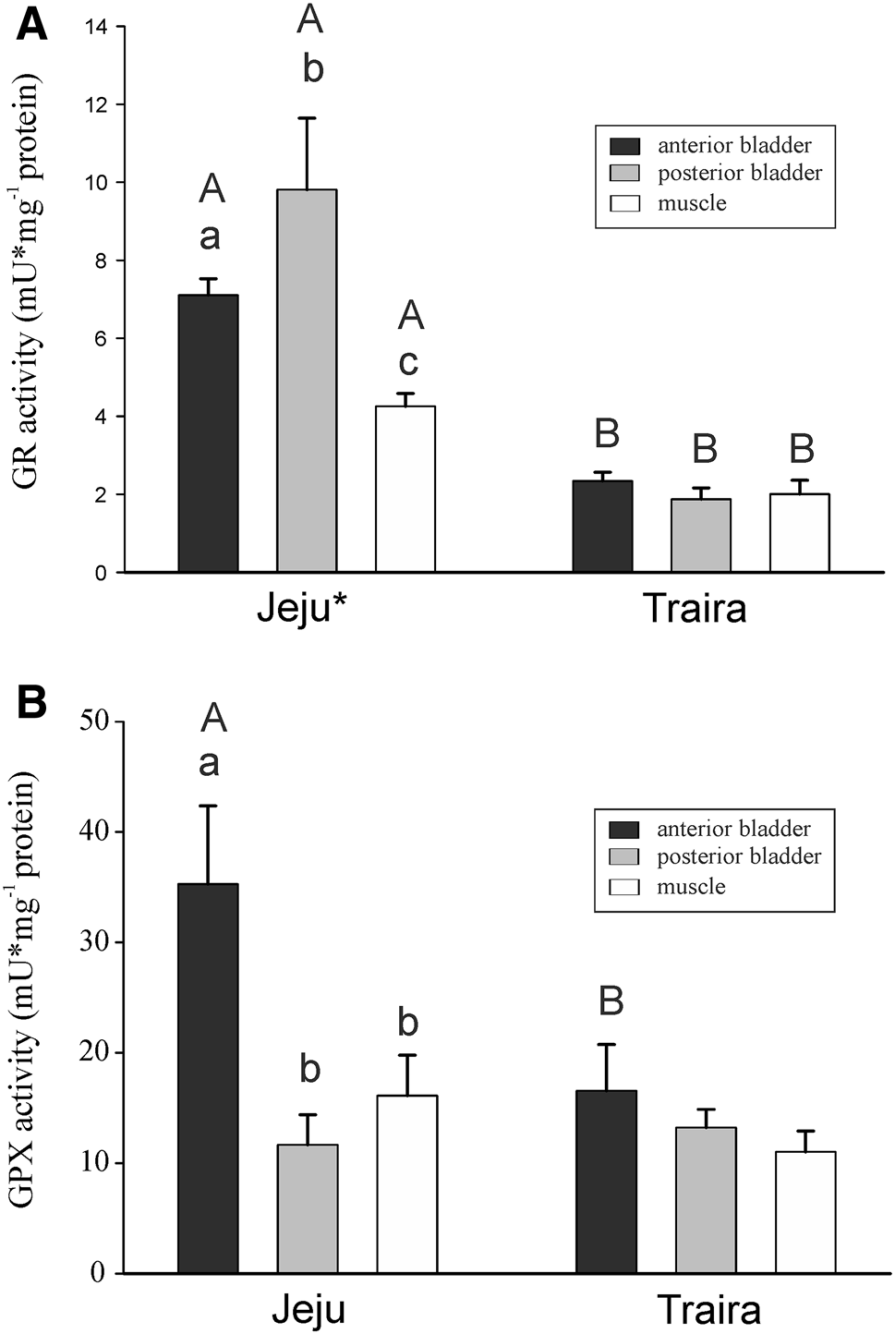
Glutathione reductase (**A**) and glutathione peroxidase (**B**) activity in mU mg^−1^ protein in anterior and posterior swimbladder tissue and in muscle tissue of jeju and of traira. *Small letters* denote significant differences between tissues within a species, *capital letters* denote significant differences between the two species, *bars* without letters are not significantly different, * asterisk* indicates significant overall difference between the two species (*N* = 8; *p* < 0.05)

Significant differences were identified between the two species with respect to GR activity (*p* < 0.001; *F*_1,14_ = 72.748). Comparing the three individual tissues GR activity was higher in jeju tissues (*p* < 0.001 for anterior and posterior swimbladder tissue; *p* < 0.05 for muscle tissue) (Fig. 6A). The highest activity was recorded in posterior swimbladder tissue of jeju, followed by the anterior swimbladder. Within jeju all three tissues were different in GR activity, muscle tissue showing the lowest activity. Within traira no difference in the GR activity was detected between muscle and swimbladder tissue.

At the species level GPx activity was not different between jeju and traira, but in the anterior part of jeju swimbladder GPx activity was higher than in anterior swimbladder tissue of traira (*p* < 0.01) (Fig. 6B). The highest GPx activity was found in anterior swimbladder tissue of the jeju with 35.3 ± 7.1 mU mg^−1^ protein (Fig. 6B), and the activity was higher than in posterior swimbladder tissue or muscle tissue of the jeju. In traira again no difference was detected in GPx activity when comparing swimbladder and muscle tissue (Fig. 6B).

## Discussion

In behavioral studies on the jeju we observed air-breathing behavior under normoxic conditions, while in previous studies air-breathing was not observed at PO_2_ values above 42.5 % air saturation (PO_2_ = 8.5 kPa) (Perry et al. 2004; Oliveira et al. 2004). In the latter studies air-breathing was only observed at lower PO_2_ values, reaching a maximum of 20 or 36 breathing events per hour at a PO_2_ of 2.3 kPa. It is quite possible that in our study jeju resorted to air-breathing at normoxia due to higher temperatures, which increase metabolic activity. Our experiments were performed in the field using water from the river at river temperature, while the other two studies were performed in the lab at 25 °C. In the present study 21.7 breaths per hour were observed at the lowest PO_2_. A much lower frequency of only 5.6 breaths per hour was observed by Juca-Chagas (Juca-Chagas 2004) at a PO_2_ of below 3.3 % air saturation (PO_2_ = 0.67 kPa). These experiments were performed at a temperature of 25 °C, which may have contributed to these lower values, but Perry et al. (2004) counted 36 breathing events per hour at a PO_2_ of 13 % air saturation (PO_2_ = 2.6 kPa) at the same temperature. Accordingly, temperature does not appear the only explanation for these differences. A stimulation of air-breathing behavior by severe hypoxia has also been reported in previous studies on the jeju (Kramer et al. 1978; Stevens and Holeton 1978). Quite surprising and unexpected was the observation that hyperoxia did not reduce air-breathing activity in the jeju. Following the hypothesis that aquatic hypoxia stimulates aerial respiration in fish (Daniels et al. 2004), it could be expected that surplus oxygen in the water will reduce air-breathing activity. This observation supported the conclusion that temperature alone was not the clue for initiation of normoxic air-breathing behavior in the jeju in our experiments, because normoxic and hyperoxic conditions were encountered at the same temperature in individual fish. The elevated oxygen supply in the water under hyperoxia therefore should have reduced the necessity to supplement aquatic oxygen uptake by breathing air. One possible explanation for the air-breathing activity at hyperoxic PO_2_ in the water could be that the high PO_2_ will increase ROS production, and in this situation breathing air with normoxic PO_2_, rather than water with hyperoxic PO_2_, may be advantageous for the tissues. In our experiments oxygen partial pressure of the water was changed, while the air space was open to the environment and not experimentally modified. Accordingly, the air taken up into the swimbladder should have been close to normoxic under all experimental conditions. Nevertheless, the jeju showed a clear behavioral response to changes in water PO_2_. Lopes et al. (2010) have shown that stimulation of branchial chemoreceptors induced air breathing behavior in the jeju and assumed that internal hypoxaemia is the primary drive to stimulate air-breathing.

The traira is not an air-breathing fish (Rantin et al. 1992), and Glass and Rantin (Glass and Rantin 2009) reported that it completely depends on gill ventilation, even under hypoxic conditions. However, our observations clearly revealed that the traira started aquatic surface respiration (ASR) when water PO_2_ dropped to very low values. This behavior, previously described for a number of tropical freshwater fish (Kramer and Mehegan 1981; Kramer and McClure 1982), can be seen as a step prior to air breathing in evolutionary terms. The fish uses the water just beneath the water surface for ventilation. Because of diffusion from air into the water, the surface layer with close contact to the air should have the highest PO_2_.

In freshwater fish using the swimbladder as an accessory air-breathing organ, the oxygen partial pressures will not be as high as in the swimbladder of marine fish swimming at depth using the bladder as buoyancy organ [see (Steen 1963; Kobayashi et al. 1990; Pelster 1997, 2009, 2011)], but they are expected to be higher than oxygen partial pressures of most tissues of purely water breathing fish. In normoxic Atlantic salmon *Salmo salar* arterial PO_2_ is lower than water PO_2_ (Kristensen et al. 2010), and in dorsal arterial blood of traira and jeju PO_2_ values of 10 and 4 kPa have been reported (Stevens and Holeton 1978; Randall et al. 1978; Perry et al. 2004), respectively. This is much lower than the average PO_2_ of 13.3 kPa reported for anterior swimbladder of jeju, and even values of up to 18 and 20 kPa have been measured in jeju swimbladder (Farrell and Randall 1978; Kramer 1978). Due to the rare uptake of air into the swimbladder of traira, which is used for buoyancy purposes and not for oxygen uptake, and the accumulation of less soluble inert gases in non-ventilated body cavities (Piiper 1965; Pelster 1997), swimbladder tissue of jeju was expected to be exposed to much higher PO_2_ values than traira swimbladder tissue. Air exposure of the facultative air-breathing fish *Heteropneustes fossilis* initially resulted in an increase in lipid peroxidation, as indicated by an increase in thiobarbituric acid reactive substances (TBARS), an increase in protein carbonylation and in H_2_O_2_ concentration in muscle tissue, indicating that the higher PO_2_ values encountered in air indeed result in an elevated ROS production (Paital 2014). Thus, exposure of fish tissues to air may stimulate ROS production, and the results of our present study clearly confirm the hypothesis that swimbladder tissue of the jeju, used as an additional air-breathing organ, has an improved antioxidative capacity in comparison to the swimbladder tissue of traira, used as buoyancy organ.

The increased antioxidative capacity extends to the concentration of small antioxidants (GSSG/GSH) as well as to the activity of enzymes involved in ROS degradation. While in the traira the GSSG/GSH concentration was similar in white muscle and in swimbladder tissue, in jeju swimbladder tissue the concentration was almost twice as high as in muscle tissue, and at the species level in the jeju the concentration was significantly higher than in the traira. Glutathione is considered to be a very important component of the antioxidant defense in cells (Storey 1996; Lushchak and Bagnyukova 2006), and an increase in GSH concentration was found in trout hepatocytes after exposure to hyperoxia (Ritola et al. 2002). Similarly, recovery from hypoxia, a situation typically connected to increased ROS production, resulted in a two-fold increase in glutathione concentration in common carp liver (Lushchak et al. 2005b).

Similar to the total glutathione concentration, at the species level the activity of catalase, GR and GPX, enzymes involved in ROS degradation, was higher in the jeju as compared to the traira. Catalase activity was significantly higher in posterior swimbladder of the jeju. An increase in the activity of catalase has been observed during recovery from hyperoxic exposure in goldfish tissues (Lushchak et al. 2005a), supporting the conclusion that the high PO_2_ values encountered in the swimbladder in the air-breathing jeju may be the cause for the elevated activity of catalase.

Looking at the activity of GR and GPx the difference between the two erythrinid species became even more obvious. Especially GR activity was 3–4 times higher in jeju swimbladder tissue, and in the anterior bladder GPx activity was also significantly elevated. Taken together with the higher GSSG/GSH concentration this suggests that the glutathione-based ROS defense is more important than the immediate ROS degradation by SOD and catalase activity. Similarly, in goldfish the responses of glutathione-dependent enzymes to different levels of oxygen exceed responses of SOD and catalase (Lushchak et al. 2001, 2005a; Bagnyukova et al. 2006). So far most studies focusing on antioxidants in fish have used liver as a tissue highly exposed to reactive oxygen species due to its metabolic activity including, for example, cytochrome P450 activity for the detoxification of xenobiotics (Lushchak et al. 2005a; Bagnyukova et al. 2006). Although the response to oxidative stress induced by changes in oxygen availability in fish tissues does not appear to be uniform (Welker et al. 2013), the consistently elevated GSSG/GSH concentrations and the elevated activities of enzymes involved in ROS defense found in the swimbladder tissue of jeju as compared to traira suggested that using the swimbladder as an air-breathing organ may be connected to special protection against ROS.

According to the vascularization, the posterior swimbladder of the jeju is the part where the oxygen is removed from the gas phase of the bladder. When gulping air the air is initially transferred into the anterior part of the bladder. Gas is then released from the posterior part of the bladder through the operculum, and the posterior bladder is refilled with gas from the anterior part of the bladder (Randall et al. 1981). Accordingly, the anterior part of the bladder is exposed to even higher PO_2_ values than the posterior part, where the uptake of oxygen results in decreasing PO_2_ values (Kramer 1978). Nevertheless, except for GR, which showed a significantly higher activity in the posterior part of the jeju swimbladder, no difference in the ROS defense capacity was detected between the two sections.

Somewhat unexpected was the observation that a higher GSH/GSSG concentration was detected in muscle tissue of jeju than in muscle of traira, and GR activity was significantly higher. Based on the assumption that higher tissue PO_2_ coincides with a higher ROS defense capacity this may indicate that muscle of tissue of jeju obtains better oxygen supply, at least during periods of intensive air-breathing.

In evolutionary terms the relation between the fish swimbladder and the tetrapod lung has frequently been discussed (Perry et al. 2001). Only recently, molecular studies revealed that the swimbladder is homologous to the mammalian lung (Zheng et al. 2011) and there are in fact several common properties in these air-filled organs. Lung function crucially depends on the presence of surfactant produced by type II pneumocytes, and surfactant is also produced by gas gland cells of the fish swimbladder (Prem et al. 2000). A detailed analysis of the surfactant composition of various fish revealed the uniform composition of surfactant from the fish swimbladder and the tetrapod lung (Daniels et al. 2004). Our present results support the hypothesis that jeju, using the swimbladder as an accessory air-breathing organ, has an improved oxidative capacity in the swimbladder tissue when compared to traira swimbladder, used as a buoyancy organ. In a recent study we could also demonstrate that in European eel during silvering, which prepares the animal for the spawning migration to the Sargasso Sea with highly elevated PO_2_ pressure in the swimbladder at depth, the ROS defense capacity is significantly improved (G. Schneebauer, R. Hanel and B. Pelster, unpublished). It therefore appears likely that the exposure of lung or swimbladder tissue to elevated PO_2_ values coincides with an enhanced antioxidative capacity of the tissues.

## References

[CR1] Ali SS, Hsiao M, Zhao HW, Dugan LL, Haddad GG, Zhou D (2012). Hypoxia-adaptation involves mitochondrial metabolic depression and decreased ROS leakage. PLoS One.

[CR2] Bagnyukova TV, Chahrak OI, Lushchak VI (2006). Coordinated response of goldfish antioxidant defenses to environmental stress. Aquatic Toxicol.

[CR3] Bemis WE, Burggren WW, Kemp NE (1987). The biology and evolution of lungfishes.

[CR4] Bradford MM (1976). A rapid and sensitive method for the quantitation of microgram quantities of protein utilizing the principle of protein-dye binding. Anal Biochem.

[CR5] Chandel NS, Budinger GRS (2007). The cellular basis for diverse responses to oxygen. Free Rad Biol Med.

[CR6] Daniels CB, Orgeig S, Sullivan LC, Ling N, Bennett MB, Schurch S, Val AL, Brauner CJ (2004). The origin and evolution of the surfactant system in fish: insights into the evolution of lungs and swim bladders. Physiol Biochem Zool.

[CR7] Diaz RJ, Breitburg DL, Richards JG, Farrell AP, Brauner CJ (2009). The hypoxic environment. Hypoxia.

[CR8] Erzurum SC, Danel C, Gillissen A, Chu CS, Trapnell BC, Crystal RG (1993). In vivo antioxidant gene expression in human airway epithelium of normal individuals exposed to 100 % O_2_. J Appl Physiol.

[CR9] Farrell AP, Randall DJ (1978). Air-breathing mechanics in two Amazonian teleosts, *Arapaima gigas* and *Hoplerythrinus unitaeniatus*. Can J Zool.

[CR10] Gilmour KM, Perry SF (1994). The effects of hypoxia, hyperoxia or hypercapnia on the acid-base disequilibrium in the arterial blood of rainbow trout. J Exp Biol.

[CR11] Glass ML, Rantin FT, Glass ML, Wood SC (2009). Gas exchange and control of respiration in air-breathing teleost fish. Cardio-respiratory control in vertebrates—evolutionary and evolutionary aspects.

[CR12] Gorr TA, Wichmann D, Hu J, Hermes-Lima M, Welker AF, Terwilliger N, Wren JF, Viney M, Morris S, Nilsson GE, Deten A, Soliz J, Gassmann M (2010). Hypoxia tolerance in animals: biology and application. Physiol Biochem Zool.

[CR13] Guppy M (2004). The biochemistry of metabolic depression: a history of perceptions. Comp Biochem Physiol Part B Biochem Molec Biol.

[CR14] Hackett NR, Heguy A, Harvey B-G, O’Connor TP, Luettich K, Flieder DB, Kaplan R, Crystal RG (2003). Variability of antioxidant-related gene expression in the airway epithelium of cigarette smokers. Am J Respir Cell Mol Biol.

[CR15] Halliwell B, Gutteridge JMC (1989). Free radicals in biology and medicine.

[CR16] Hermes-Lima M, Storey KB (2004). Oxygen in biology and biochemistry: the role of free radicals. Functional metabolism: regulation and adaptation.

[CR17] Hermes-Lima M, Storey JM, Storey KB (1998). Antioxidant defenses and metabolic depression: the hypothesis of preparation for oxidative stress in land snails. Comp Biochem Physiol Part B.

[CR18] Ho JC, Zheng S, Comhair SAA, Farver C, Erzurum SC (2001). Differential expression of manganese superoxide dismutase and catalase in lung cancer. Cancer Res.

[CR19] Juca-Chagas R (2004). Air breathing of the neotropical fishes *Lepidosiren paradoxa, Hoplerythrinus unitaeniatus* and *Hoplosternum littorale* during aquatic hypoxia. Comp Biochem Physiol Part A Molec Integ Physiol.

[CR20] Kobayashi H, Pelster B, Scheid P (1990). CO_2_ back-diffusion in the rete aids O_2_ secretion in the swimbladder of the eel. Respir Physiol.

[CR21] Kramer DL (1978). Ventilation of the respiratory gas bladder in *Hoplerythrinus unitaeniatus* (Pisces, Characoidei, Erythrinidae). Can J Zool.

[CR22] Kramer DL, McClure M (1982). Aquatic surface respiration, a widespread adaptation to hypoxia in tropical freshwater fishes. Env Biol Fish.

[CR23] Kramer DL, Mehegan JP (1981). Aquatic surface respiration, an adaptive response to hypoxia in the guppy, *Poecilia reticulata* (Pisces, Poeciliidae). Env Biol Fish.

[CR24] Kramer DL, Lindsey CC, Moodie GEE, Stevens ED (1978). The fishes and the aquatic environment of the central Amazon basin, with particular reference to respiratory patterns. Can J Zool.

[CR25] Kristensen T, Rosseland BO, Kiessling A, Djordevic B, Massabau JC (2010). Lack of arterial PO_2_ downregulation in Atlantic salmon (*Salmo salar L*.) during long-term normoxia and hyperoxia. Fish Physiol Biochem.

[CR26] Li C, Jackson RM (2002). Reactive species mechanisms of cellular hypoxia-reoxygenation injury. Amer J Physiol Cell Physiol.

[CR27] Lopes JM, de Lima Boijink C, Florindo LH, Leite CAC, Kalinin AL, Milsom WK, Rantin FT (2010). Hypoxic cardiorespiratory reflexes in the facultative air-breathing fish jeju (*Hoplerythrinus unitaeniatus*): role of branchial O_2_ chemoreceptors. J Comp Physiol B.

[CR28] Lushchak VI (2011). Environmentally induced oxidative stress in aquatic animals. Aquatic Toxicol.

[CR29] Lushchak VI (2014). Free radicals, reactive oxygen species, oxidative stress and its classification. Chem -Biol Interact.

[CR30] Lushchak VI, Bagnyukova TV (2006). Effects of different environmental oxygen levels on free radical processes in fish. Comp Biochem Physiol Part B Biochem Molec Biol.

[CR31] Lushchak VI, Bagnyukova TV (2007). Hypoxia induces oxidative stress in tissues of a goby, the rotan *Perccottus glenii*. Comp Biochem Physiol Part B Biochem Molec Biol.

[CR32] Lushchak VI, Lushchak LP, Mota AA, Hermes-Lima M (2001). Oxidative stress and antioxidant defenses in goldfish *Carassius auratus* during anoxia and reoxygenation. Amer J Physiol Reg Integ Comp Physiol.

[CR33] Lushchak VI, Bagnyukova TV, Husak VV, Luzhna LI, Lushchak OV, Storey KB (2005). Hyperoxia results in transient oxidative stress and an adaptive response by antioxidant enzymes in goldfish tissues. Int J Biochem Cell Biol.

[CR34] Lushchak VI, Bagnyukova TV, Lushchak OV, Storey JM, Storey KB (2005). Hypoxia and recovery perturb free radical processes and antioxidant potential in common carp (*Cyprinus carpio*) tissues. Int J Biochem Cell Biol.

[CR35] McCord JM, Fridovich I (1969). Superoxide dismutase. An enzymic function for erythrocuprein (hemocuprein)*. J Biol Chem.

[CR36] Morris SM, Albright JT (1981). Superoxide dismutase, catalase, and glutathione peroxidase in the swim bladder of the physoclistous fish, *Opsanus tau* L. Cell Tiss Res.

[CR37] Morris SM, Albright JT (1984). Catalase, glutathione peroxidase, and superoxide dismutase in the rete mirabile and gas gland epithelium of six species of marine fishes. J Exp Zool.

[CR38] Muusze B, Marcon J, Van den Thillart G, Almeida-Val V (1998). Hypoxia tolerance of Amazon fish respirometry and energy metabolism of the cichlid *Astronotus ocellatus*. Comp Biochem Physiol Part A.

[CR39] Oliveira RD, Lopes JM, Sanches JR, Kalinin AL, Glass ML, Rantin FT (2004). Cardiorespiratory responses of the facultative air-breathing fish jeju, *Hoplerythrinus**unitaeniatus* (Teleostei, Erythrinidae), exposed to graded ambient hypoxia. Comp Biochem Physiol Pt A: Molec Integ Physiol.

[CR40] Paital B (2014). Modulation of redox regulatory molecules and electron transport chain activity in muscle of air breathing fish *Heteropneustes fossilis* under air exposure stress. J Comp Physiol B.

[CR41] Pelster B, Randall DJ, Farrell AP (1997). Buoyancy at depth. Deep-Sea Fish.

[CR42] Pelster B, Glass ML, Wood SC (2009). Buoyancy control in aquatic vertebrates. Cardio-respiratory control in vertebrates.

[CR43] Pelster B, Farrell AP (2011). Swimbladder function and buoyancy control in fishes. Encyclopedia of fish physiology: from genome to environment.

[CR44] Pelster B (2015). Swimbladder function and the spawning migration of the European eel *Anguilla anguilla*. Front Physiol.

[CR45] Perry SF, Wilson RJA, Straus C, Harris MB, Remmers JE (2001). Which came first, the lung or the breath?. Comp Biochem. Physiol Part A.

[CR46] Perry SF, Reid SG, Gilmour KM, Boijink CL, Lopes JM, Milsom WK, Rantin FT (2004). A comparison of adrenergic stress responses in three tropical teleosts exposed to acute hypoxia. Amer J Physiol Reg Integ Comp Physiol.

[CR47] Piiper J (1965) Physiological equilibria of gas cavities in the body. In: Fenn WO, Rahn H (eds) Handbook of physiology, respiration, American Physiological Society, Bethesda, Maryland, vol 2, pp 1205–1218

[CR48] Piiper J, Humphrey HT, Rahn H (1962). Gas composition of pressurized, perfused gas pockets and the fish swim bladder. J Appl Physiol.

[CR49] Prem C, Salvenmoser W, Würtz J, Pelster B (2000). Swimbladder gas gland cells produce surfactant: in vivo and in culture. Am J Physiol Reg Integ Comp Physiol.

[CR50] Randall DJ, Farrell AP, Haswell MS (1978). Carbon dioxide excretion in the jeju, *Hoplerythrinus unitaeniatus*, a facultative air-breathing teleost. Can J Zool.

[CR51] Randall DJ, Burggren WW, Farrell AP, Haswell MS (1981). The evolution of air-breathing in vertebrates.

[CR52] Rantin FT, Kalinin AL, Glass ML, Fernandes MN (1992). Respiratory responses to hypoxia in relation to mode of life of two erythrinid species (*Hoplias malabaricus* and *Hoplias**lacerdae*). J Fish Biol.

[CR53] Ritola O, Tossavainen K, Kiuru T, Lindström-Seppä P, Mölsä H (2002). Effects of continuous and episodic hyperoxia on stress and hepatic glutathione levels in one-summer-old rainbow trout (*Oncorhynchus mykiss*). J Appl Ichthyol.

[CR54] Sies H (2015). Oxidative stress: a concept in redox biology and medicine. Redox Biol.

[CR55] Steen JB (1963). The physiology of the swimbladder in the eel *Anguilla vulgaris*. III. The mechanism of gas secretion. Acta Physiol Scand.

[CR56] Stevens ED, Holeton GF (1978). The partitioning of oxygen uptake from air and from water by erythrinids. Can J Zool.

[CR57] Storey KB (1996). Oxidative stress: animal adaptations in nature. Braz J Med Biol Res.

[CR58] St-Pierre J, Tattersall GJ, Boutilier RG (2000). Metabolic depression and enhanced O_2_ affinity of mitochondria in hypoxic hypometabolism. Am J Physiol Reg Integ Comp Physiol.

[CR59] Summarwar S, Verma S (2012). Study of biomarkers of physiological defense against reactive oxygen species during environmental stress. Int J Life Sc Bt Pharm Res.

[CR60] Tripathi RK, Mohindra V, Singh A, Kumar R, Mishra RM, Jena JK (2013). Physiological responses to acute experimental hypoxia in the air-breathing Indian catfish, *Clarias batrachus* (Linnaeus, 1758). J Biosci.

[CR61] Val AL, Almeida-Val VMF (1995). Fishes of the Amazon and their environment.

[CR62] van Ginneken V, van den Thillart G (2009). Metabolic depression in fish measured by direct calorimetry: a review. Thermochim Acta.

[CR63] Welker AF, Moreira DC, Campos EG, Hermes-Lima M (2013). Role of redox metabolism for adaptation of aquatic animals to drastic changes in oxygen availability. Comp Biochem Physiol Part A Molec Integ Physiol.

[CR64] Wolf K (1963). Physiological salines for freshwater teleosts. Prog Fish-Cult.

[CR65] Zheng W, Wang Z, Collins JE, Andrews RM, Stemple D, Gong Z (2011). Comparative transcriptome analyses indicate molecular homology of zebrafish swimbladder and mammalian lung. PLoS One.

